# Antibacterial Activity and Optimal Treatment of Ceftazidime-Avibactam and Aztreonam-Avibactam Against Bloodstream Infections Caused by Carbapenem-Resistant *Klebsiella pneumoniae*

**DOI:** 10.3389/fphar.2021.771910

**Published:** 2021-12-14

**Authors:** Wei Yu, Yunbo Chen, Ping Shen, Jinru Ji, Chaoqun Ying, Zhiying Liu, Luying Xiong, Yunqing Qiu, Yonghong Xiao

**Affiliations:** State Key Laboratory for Diagnosis and Treatment of Infectious Diseases, National Clinical Research Center for Infectious Diseases, Collaborative Innovation Center for Diagnosis and Treatment of Infectious Diseases, The First Affiliated Hospital, Zhejiang University School of Medicine, Hangzhou, China

**Keywords:** carbapenem-resistant *Klebsiella pneumoniae*, PK/PD, intermittent infusion, two-step infusion, Monte Carlo simulation

## Abstract

**Objectives:** This work was to investigate the activity and optimal treatments of ceftazidime-avibactam (CZA) and aztreonam-avibactam (AZA) against bloodstream infections caused by carbapenem resistant *Klebsiella pneumoniae* (BSIs-CRKP).

**Methods:** A total of 318 nonduplicate BSIs-CRKP isolates were collected from Blood Bacterial Resistant Investigation Collaborative System (BRICS) program. The minimum inhibitory concentration (MIC) of CZA and AZA were determined by agar dilution method. Carbapenemase genes and multilocus sequence typing were amplified by PCR. Monte Carlo simulation (MCS) was conducted to calculate cumulative fraction of response (CFR) of different CZA or AZA administrations.

**Results:** The MIC_90_ of CZA and AZA were 128/4 and 1/4 mg/L, respectively. There are 87.4 and 3.5% isolates carried *bla*_KPC-2_ and *bla*_NDM-1_. A total of 68 ST types were identified and 29 novel ST types. ST11 accounted for 66.6%. Further MCS showed CFR of CZA using two-step infusion therapy (rapid first-step 0.5 h infusion and slow second-step 3 h infusion, TSIT) (2.5 g 0.5 h, 3.75 g every 8 h with 3 h infusion and 3.75 g 0.5 h, 2.5 g every 8 h with 3 h infusion) was above 89%. The CFR of AZA with TSIT was above 96%.

**Conclusion:** TSIT with sufficient pharmacokinetic conditions could be useful for enhancing the therapeutic efficacy of CZA and AZA against BSIs-CRKP.

## Introduction

Carbapenem-resistant Enterobacteriaceae (CRE), especially for bloodstream infections caused by carbapenem-resistant *Klebsiella pneumoniae* (BSIs-CRKP), have become a major healthcare burden in the 21st century. The incidence of BSIs-CRKP worldwide remained uptrend so far, especially in Europe and Asia Pacific (Keepers et al., 2014). The treatments of CRE usually required combination therapy before 2014 (Morrill et al., 2015; Doi, 2019). However, the toxicity of some combinations is obvious, resulting in adverse effect during clinical treatment. Although several combination regimens have been optimized, there are still no uniform standards for antibiotics against BSIs-CRKP. Since 2014, the development of new antibiotics brought new opportunities for treatments of CRE. The existing approved novel antibiotics against CRE infections included ceftazidime-avibactam (CZA), meropenem-vaborbactam, imipenem/cilastatin-relebactam, plazomicin and eravacycline (Papp-Wallace, 2019). However, none of these β-lactamase-inhibitor combinations showed activity against all entire carbapenemase, especially for metallo-β-lactamase (MBL) (Biagi et al., 2019). Fortunately, recent novel antibiotic aztreonam-avibactam (AZA) represented remarkable progress in the treatment of MBL- or other β-lactamases-producing CRE.

CZA and AZA are time-dependent antibiotics for which the time of free plasma concentration above the minimum inhibitory concentration (MIC) (%fT > MIC) is the best predictor of efficacy (Keepers et al., 2014; Cornely et al., 2020). Traditional 0.5 h infusion therapy (TIT) is the most common way of antibacterial prescription used in clinic (Shiu et al., 2013). However, the serum concentration of several time-dependent antibiotics dropped below the MIC before the next scheduled intermittent infusion during TIT (Lipman et al., 2001). Therefore, to prolong %fT > MIC and improve the efficacy, alternative dosing strategies have been studied, such as prolonged 3 h infusion therapy (PIT) and two-step infusion therapy (rapid first-step 0.5 h infusion and slow second-step 3 h infusion, TSIT) (Tamma et al., 2011). However, clinical experience of new antibiotics and comparative data among new agents are lacking. Therefore, we evaluated the activity of CZA and AZA against BSIs-CRKP *in vitro* and optimize treatments using Monte Carlo simulation (MCS), in order to provide theoretical basis for clinical treatment.

## Materials and Methods

### Bacterial Isolates

A total of non-duplicate 318 BSIs-CRKP isolates were collected by Blood Bacterial Resistant Investigation Collaborative System (BRICS) program from 34 hospitals in 2019 in China (Wei et al., 2021). Multi-locus sequence typing (MLST) was performed using the scheme of Pasteur database (Supplementary Table S1). Multiple sequence alignments were performed using PHYLOViZ 2.0. The phylogenetic tree was visualized and edited using Interactive Tree of Life (iTOL) (Letunic and Bork, 2019).

The minimum inhibitory concentration (MIC) of 24 antibiotics (including CZA and AZA) were determined in our previous study (Wei et al., 2021). Carbapenemase-producing isolates were further identified using modified Hodge test according to Clinical and Laboratory Standards Institute (CLSI) guidelines (Clinical and Laboratory S, 2021). Hypervirulent phenotype was performed with the string test (Shon et al., 2013). Carbapenemase genes were routinely amplified by PCR (Poirel et al., 2011).

### Pharmacokinetics Parameters and Pharmacokinetics/Pharmacodynamics Target

CZA and AZA displayed time-dependent bactericidal effect. The results of %fT > MIC were calculated as a previous study (Yu et al., 2017; Eguchi et al., 2010). The details of PK/PD equations were shown in Supplementary Table S2. The main PK parameters, including volume of distribution, clearance rate, and free drug fraction, referred to published PK studies of CZA and AZA (Vinks et al., 2007; Stein et al., 2019; Cornely et al., 2020). The PK/PD indexes of %fT > MIC > 50% and %fT > MIC > 60% were used as the target for CZA and AZA.

### Monte Carlo Simulation

A total of 22 regimens of CZA (4:1) and 36 regimens of AZA (3.65:1) were investigated (Table 1). The doses were only expressed as ceftazidime and aztreonam for CZA and AZA in the present study.

**TABLE 1:** Regimens of CZA and AZA.

Antibiotics	TIT	PIT	TSIT
CZA (4:1) (only CAZ shown)	1 g q8h	1 g q8h 3 h	1 g 0.5 h + 1 g q8h 3h, 2 g 0.5 h + 1 g q8h 3 h
	2 g q8h	2 g q8h 3 h	3 g 0.5 h + 1 g q8h 3h, 1 g 0.5 h + 1 g q6h 3 h
	3 g q8h	3 g q8h 3 h	2 g 0.5 h + 1 g q6h 3h, 1 g 0.5 h + 2 g q8h 3 h
	1 g q6h	1 g q6h 3 h	1 g 0.5 h + 3 g q8h 3h, 2 g 0.5 h + 2 g q8h 3 h
	2 g q6h	2 g q6h 3 h	2 g 0.5 h + 3 g q8h 3h, 3 g 0.5 h + 2 g q8h 3 h
	—	—	1 g 0.5 h + 2 g q6h 3h, 2 g 0.5 h + 2 g q6h 3 h
AZA (3.65:1) (only ATM shown)	0.5 g q8h	0.5 g q8h 3 h	0.5 g 0.5 h + 0.5 g q8h 3 h, 0.5 g 0.5 h + 1 g q8h 3 h
	1 g q8h	1 g q8h 3 h	0.5 g 0.5 h + 1.5 g q8h 3 h, 0.5 g 0.5 h + 2 g q8h 3 h
	1.5 g q8h	1.5 g q8h 3 h	0.5 g 0.5 h + 0.5 g q6h 3 h, 0.5 g 0.5 h + 1 g q6h 3 h
	2 g q8h	2 g q8h 3 h	0.5 g 0.5 h + 1.5 g q6h 3 h, 0.5 g 0.5 h + 2 g q6h 3 h
	2.5 g q8h	2.5 g q8h 3 h	1 g 0.5 h + 1 g q8h 3 h, 1 g 0.5 h + 1.5 g q8h 3 h
	0.5 g q6h	0.5 g q6h 3 h	1 g 0.5 h + 2 g q8h 3 h, 1.5 g 0.5 h + 1.5 g q8h 3 h
	1 g q6h	1 g q6h 3 h	2 g 0.5 h + 2 g q8h 3 h, 1 g 0.5 h + 1 g q6h 3 h
	1.5 g q6h	1.5 g q6h 3 h	1 g 0.5 h + 1.5 g q6h 3 h, 1 g 0.5 h + 2 g q6h 3 h
	2 g q6h	2 g q6h 3 h	1.5 g 0.5 h + 1.5 g q6h 3 h, 2 g 0.5 h + 2 g q6h 3 h

CAZ, ceftazidime; CZA, ceftazidime-avibactam; ATM, aztreonam; AZA, aztreonam-avibactam; h, hour; TIT, traditional 0.5 h infusion therapy; PIT, prolonged 3 h infusion therapy; TSIT, two-step infusion therapy (rapid first-step 0.5 h infusion and slow second-step 3 h infusion); q8h, every 8 hours; q6h, every 6 hours.

A 10,000-subject MCS was performed to calculate the probability of target attainment (PTA) and cumulative fraction of response (CFR) of each dosing regimen against BSIs-CRKP using Crystal Ball software (version 11.1.2.4; Oracle). Plasma clearance rate and volume of distribution were assumed to follow log-normal distribution. The definition assumption of free drug fraction was used as uniform distribution.

The PTA value of each drug regimen was considered to be adequate when a target of ≥90% was reached. An optimal regimen was defined as achieving ≥90% CFR against a population of organisms whereas a CFR between 80 and 90% was associated with moderate probabilities of success (Bradley et al., 2003).

## Results

### Geographical Distribution and MLST of BSIs-CRKP

A total of 318 BSIs-CRKP were collected from 34 hospitals, including 32 tertiary hospitals and 2 secondary graded hospitals. All isolates were from East China (EC) (*n* = 262), Central China (CC) (*n* = 30), Northeast China (NE) (*n* = 9), Northwest China (NW) (*n* = 3), Southwest China (SW) (*n* = 14), respectively (Supplementary Figure S1).

**FIGURE 1:**
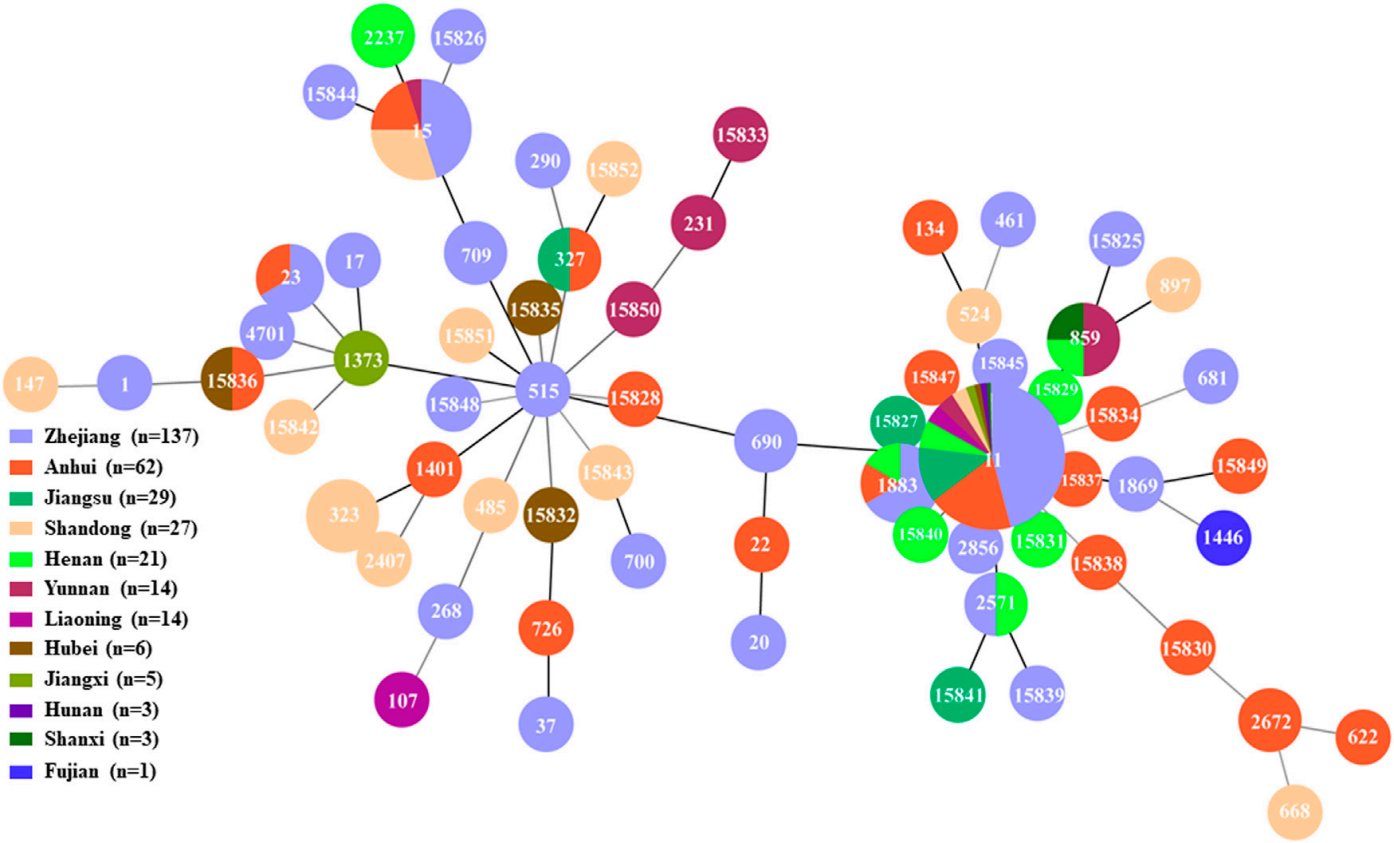
Minimum spanning tree of 318 BSIs-CRKP isolates.

MLST revealed 68 different STs, and 29 novel STs that are not registered in the *Klebsiella* Pasteur MLST database. ST11 was the most abundant type (212 isolates, 66.6%), followed by ST15 (20 isolates, 6.3%), ST1883 (6 isolates, 1.9%), ST323 (4 isolates, 1.3%) and ST859 (6 isolates, 1.9%). The genetic relationships showed ST11 was closely related to ST690, ST1883, ST2856, ST15827, ST15829, ST15831, ST15837, and ST15845 (Figure 1). The types of STs exhibited a diversity in EC. Cluster analysis based on the conserved housekeeping gene classified 318 isolates into three distinct evolutionary lineages (Supplementary Figure S2).

### MICs Distribution of CZA and AZA and Resistance Genes

The MICs distribution of CZA and AZA were shown in Table 2. Recent clinical breakpoint of AZA has not been approved. MIC_90_ of CZA and AZA were 128/4 and 1/4 mg/L, respectively.

**TABLE 2:** The MICs distribution of CZA and AZA in 318 BSI-CRKP isolates.

MIC (mg/L)	CZA (N/%)	AZA (N/%)
0.015	—	2 (0.63%)
0.03	—	2 (0.63%)
0.06	—	11 (3.46%)
0.125	—	24 (7.55%)
0.25	5 (1.57%)	44 (13.84%)
0.5	8 (2.52%)	134 (42.14%)
1	33 (10.38%)	76 (23.9%)
2	107 (33.65%)	11 (3.46%)
4	113 (35.53%)	3 (0.94%)
8	12 (3.77%)	1 (0.31%)
16	2 (0.63%)	0
32	1 (0.31%)	2 (0.63%)
64	2 (0.63%)	2 (0.63%)
128	35 (11.01%)	3 (0.94%)
256	—	3 (0.94%)
MIC_50_	4/4	0.5/4
MIC_90_	128/4	1/4

MIC, minimum inhibitory concentration; CZA, ceftazidime-avibactam; AZA, aztreonam-avibactam; N, number; —, no data.

**FIGURE 2:**
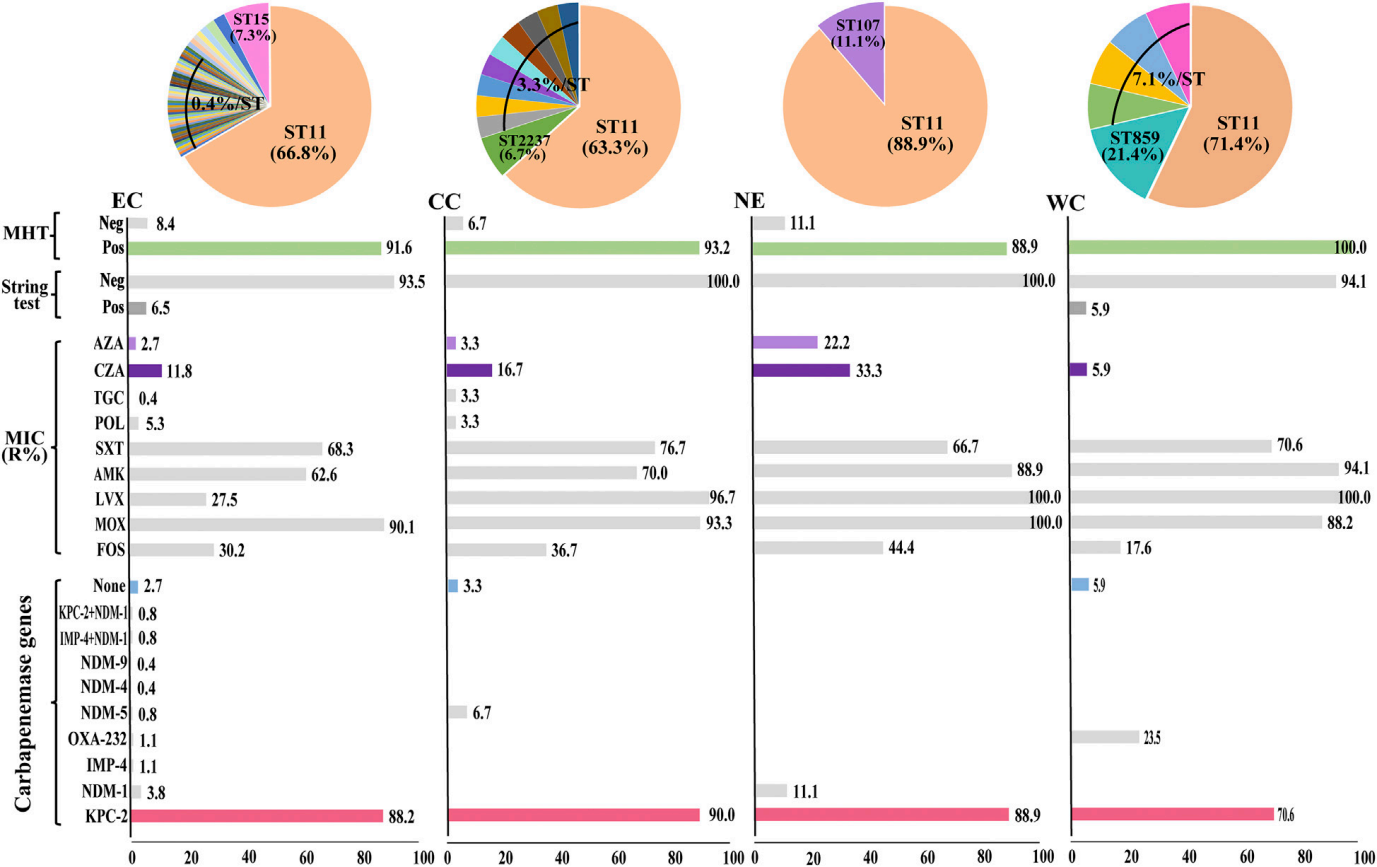
Diversity of bacterial factors in 318 BSIs-CRKP isolates. The pie charts indicate the proportions of major STs. The bar charts indicate the proportion of isolates for each variable. EC, East China; CC, Central China; NE, Northeast China; WC, West China. MHT, modified Hodge test.

The positive rates of Modified Hodge testing and string test were 92.4% (294/318) and 5.6% (18/318), respectively. There were 278 isolates (87.4%) and 11 isolates (3.5%) positive for *bla*_KPC-2_ and *bla*_NDM-1_ (Figure 2). Five isolates were coexistence of two carbapenemase genes. Two isolate was coexistence of *bla*_IMP-4_ and *bla*_NDM-1_, another three isolates were carrying both *bla*_KPC-2_ and *bla*_NDM-1_. In addition, eight isolates were not detected any carbapenemase genes. Thirteen isolates with positive string test were belong to ST11.

### Monte Carlo Stimulation

**FIGURE 3:**
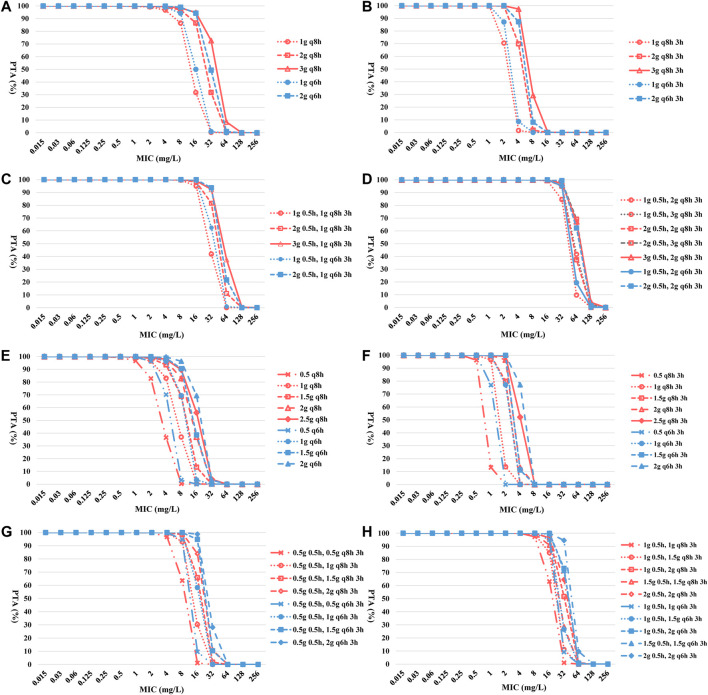
PTA-MIC curves of CZA and AZA at different simulated regimens. PTA, probability of target attainment; MIC, minimum inhibitory concentration; q8h, every 8 hours; q6h, every 6 hours. **(A)** TIT of CZA; **(B)** PIT of CZA; **(C)** and **(D)** TSIT of CZA; **(E)** TIT of AZA; **(F)** PIT of AZA; **(G)** and **(H)** TSIT of AZA.

The CFR for CZA of TIT was lower than PIT and TSIT (Figures 3A–D and Table 3). PTA reached ≥90% among CZA 2 g every 8 hours (q8h) or every 6 hours (q6h) against isolates with MICs ≤8 and ≤16 mg/L. However, none of the simulated TIT regimens achieved >90% CFR. MIC at 32 mg/L was used to calculate the PTA of eight CZA TSIT dosing regimens reaching ≥90%. Furthermore, the first-step CZA 2 g 0.5 h, followed by maintenance doses of 3 g q8h 3 h infusion or CZA 3 g 0.5 h, followed by 2 g q8h 3 h infusion displayed the highest CFR (≥89%) (Table 3).

**TABLE 3:** The PTA and CFR of different dosage regimens of CZA against 318 BSIs-CRKP isolates.

Infusion methods	Regimens (only CAZ shown	PTA of different MIC (mg/L)											CFR (%)
		0.25	0.5	1	2	4	8	16	32	64	128	256	
TIT	1 g q8h	99.98	99.87	99.65	99.18	96.66	86.46	31.69	0.30	0	0	0	85.61
	2 g q8h	99.98	99.97	99.87	99.59	99.04	96.98	86.57	31.76	0.33	0	0	87.46
	3 g q8h	99.97	99.96	99.88	99.79	99.53	98.47	94.82	72.66	8.52	0	0	87.99
	1 g q6h	100	99.97	99.93	99.8	99.12	94.24	49.91	0.85	0	0	0	87.13
	2 g q6h	100	100	99.97	99.9	99.81	98.94	94.29	49.34	0.95	0	0	88.03
PIT	1 g q8h 3 h	100	99.99	99.78	70.53	1.56	0	0	0	0	0	0	38.73
	2 g q8h 3 h	100	100	100	99.79	69.88	1.82	0	0	0	0	0	72.95
	3 g q8h 3 h	100	100	100	99.95	97.41	29.22	0.14	0	0	0	0	83.82
	1 g q6h 3 h	100	100	99.98	87.05	8.64	0	0	0	0	0	0	46.83
	2 g q6h 3 h	100	100	100	99.98	87.29	8.12	0	0	0	0	0	79.43
TSIT	1 g 0.5 h, 1 g q8h 3 h	100	100	100	100	99.95	99.64	94.94	41.84	0	0	0	88.12
	2 g 0.5 h, 1 g q8h 3 h	100	100	100	99.99	99.98	99.78	98.2	81.54	11.08	0	0	88.34
	3 g 0.5 h, 1 g q8h 3 h	100	100	100	100	99.97	99.85	99.05	**92.42**	37.55	0.36	0	88.59
	1 g 0.5 h, 1 g q6h 3 h	100	100	100	100	100	100	99.32	62.51	0.83	0	0	88.24
	2 g 0.5 h, 1 g q6h 3 h	100	100	100	100	100	100	99.91	**93.67**	21.68	0	0	88.48
	1 g 0.5 h, 2 g q8h 3 h	100	100	100	100	100	99.93	99.29	84.56	9.78	0	0	88.37
	1 g 0.5 h, 3 g q8h 3 h	100	100	100	100	100	99.99	99.80	**95.32**	41.66	0	0	88.61
	2 g 0.5 h, 2 g q8h 3 h	100	100	100	100	99.99	99.97	99.74	**95.03**	37.39	0	0	88.57
	2 g 0.5 h, 3 g q8h 3 h	100	100	100	100	100	100	99.86	**98.5**	69.11	2.06	0	**89.02**
	3 g 0.5 h, 2 g q8h 3 h	100	100	100	100	100	100	99.72	**97.34**	67.29	3.96	0	**89.21**
	1 g 0.5 h, 2 g q6h 3 h	100	100	100	100	100	100	99.99	**95.3**	19.42	0	0	88.47
	2 g 0.5 h, 2 g q6h 3 h	100	100	100	100	100	100	100	**99.3**	62.27	1.44	0	88.91

MIC, minimum inhibitory concentration; CAZ, ceftazidime; CZA, ceftazidime-avibactam; TIT, traditional 0.5 h infusion therapy; PIT, prolonged 3 h infusion therapy; TSIT, two-step infusion therapy (rapid first-step 0.5 h infusion and slow second-step 3 h infusion); q8h, every 8 hours; q6h, every 6 hours; PTA, probability of target attainment; CFR, cumulative fraction of response. Bold values means PTA > 90% when MIC of CZA = 16 mg/L and the maximum of CFR.

Except PIT (0.5 g q8h 3 h infusion or 0.5 g q6h 3 h infusion), other simulated regimens obtained more than 95% of PTA for AZA against BSIs-CRKP isolates. Similar to CZA, the CFR of different AZA TSIT regimens was higher than TIT and PIT (Figures 3E–H and Table 4). Among AZA TSIT regimens, PTA of ten strategies yielded >90% against BSIs-CRKP isolates with MIC = 16 mg/L. All simulated TSIT of AZA reached >96% of CFR.

**TABLE 4:** The PTA and CFR of different dosage regimens of AZA against 318 BSIs-CRKP isolates.

Infusion methods	Regimens (only ATM shown	PTA of different MIC (mg/L)															CFR (%)
		0.015	0.03	0.06	0.125	0.25	0.5	1	2	4	8	16	32	64	128	256	
TIT	0.5 g q8h	100	100	100	99.97	99.89	99.28	96.66	82.78	36.47	0.36	0	0	0	0	0	94.87
	1 g q8h	100	100	100	99.98	99.98	99.85	99.22	96.5	83.00	36.91	0.57	0	0	0	0	96.13
	1.5 g q8h	100	100	100	100	99.97	99.89	99.6	98.61	93.42	68.75	13.30	0	0	0	0	96.51
	2 g q8h	100	100	100	100	100	99.98	99.85	99.34	96.48	82.94	36.98	0.34	0	0	0	96.71
	2.5 g q8h	100	100	100	100	100	99.98	99.9	99.56	97.82	89.64	56.01	3.81	0	0	0	96.78
	0.5 g q6h	100	100	100	100	100	100	99.59	96.38	70.15	3.22	0	0	0	0	0	96.06
	1 g q6h	100	100	100	100	100	99.98	99.97	99.53	96.19	69.33	3.30	0	0	0	0	96.70
	1.5 g q6h	100	100	100	100	100	100	99.98	99.88	98.72	90.44	38.59	0.07	0	0	0	96.81
	2 g q6h	100	100	100	100	100	100	99.98	99.94	99.66	96.28	69.38	3.24	0	0	0	96.86
PIT	0.5 g q8h 3 h	100	100	100	100	99.93	96.17	13.24	0	0	0	0	0	0	0	0	69.79
	1 g q8h 3 h	100	100	100	100	100	99.93	96.42	13.71	0	0	0	0	0	0	0	91.74
	1.5 g q8h 3 h	100	100	100	100	100	100	99.55	79.87	0	0	0	0	0	0	0	94.81
	2 g q8h 3 h	100	100	100	100	100	100	99.92	96.5	13.03	0	0	0	0	0	0	95.59
	2.5 g q8h 3 h	100	100	100	100	100	100	100	99.14	52.13	0	0	0	0	0	0	96.07
	0.5 g q6h 3 h	100	100	100	100	100	100	77.01	0	0	0	0	0	0	0	0	86.66
	1 g q6h 3 h	100	100	100	100	100	100	100	77.14	0	0	0	0	0	0	0	94.82
	1.5 g q6h 3 h	100	100	100	100	100	100	100	99.89	11.22	0	0	0	0	0	0	95.71
	2 g q6h 3 h	100	100	100	100	100	100	100	100	77.13	0	0	0	0	0	0	96.34
TSIT	0.5 g 0.5 h, 0.5 g q8h 3 h	100	100	100	100	100	100	100	99.85	96.75	63.51	1.13	0	0	0	0	96.71
	0.5 g 0.5 h, 1 g q8h 3 h	100	100	100	100	100	100	100	99.99	99.83	93.03	30.27	0	0	0	0	96.84
	0.5 g 0.5 h, 1.5 g q8h 3 h	100	100	100	100	100	100	100	100	99.93	98.41	65.81	1.75	0	0	0	96.87
	0.5 g 0.5 h, 2 g q8h 3 h	100	100	100	100	100	100	100	100	99.97	99.47	83.97	10.79	0	0	0	96.93
	0.5 g 0.5 h, 0.5 g q6h 3 h	100	100	100	100	100	100	100	100	99.97	94.70	9.61	0	0	0	0	96.84
	0.5 g 0.5 h, 1 g q6h 3 h	100	100	100	100	100	100	100	100	100	99.92	58.27	0.10	0	0	0	96.84
	0.5 g 0.5 h, 1.5 g q6h 3 h	100	100	100	100	100	100	100	100	100	100	**94.76**	10.37	0	0	0	96.93
	0.5 g 0.5 h, 2 g q6h 3 h	100	100	100	100	100	100	100	100	100	100	**98.81**	28.16	0	0	0	97.04
	1 g 0.5 h, 1 g q8h 3 h	100	100	100	100	100	100	100	100	99.9	97.32	63.14	0.99	0	0	0	96.86
	1 g 0.5 h, 1.5 g q8h 3 h	100	100	100	100	100	100	100	100	100	98.95	85.05	11.13	0	0	0	96.93
	1 g 0.5 h, 2 g q8h 3 h	100	100	100	100	100	100	100	100	100	99.90	**96.44**	51.61	0.34	0	0	97.19
	1.5 g 0.5 h, 1.5 g q8h 3 h	100	100	100	100	100	100	100	100	99.97	99.50	**90.76**	28.52	0	0	0	97.04
	2 g 0.5 h, 2 g q8h 3 h	100	100	100	100	100	100	100	100	99.99	99.87	**96.38**	64.46	1.1	0	0	97.27
	1 g 0.5 h, 1 g q6h 3 h	100	100	100	100	100	100	100	100	100	100	**94.94**	9.23	0	0	0	96.92
	1 g 0.5 h, 1.5 g q6h 3 h	100	100	100	100	100	100	100	100	100	100	**99.22**	26.55	0	0	0	97.03
	1 g 0.5 h, 2 g q6h 3 h	100	100	100	100	100	100	100	100	100	100	**99.87**	73.1	0.7	0	0	97.32
	1.5 g 0.5 h, 1.5 g q6h 3 h	100	100	100	100	100	100	100	100	100	100	**99.70**	71.48	0.14	0	0	97.31
	2 g 0.5 h, 2 g q6h 3 h	100	100	100	100	100	100	100	100	100	100	**99.96**	**94.50**	9.64	0	0	97.52

MIC, minimum inhibitory concentration; ATM, aztreonam; AZA, aztreonam-avibactam; TIT, traditional 0.5 h infusion therapy; PIT, prolonged 3 h infusion therapy; TSIT, two-step infusion therapy (rapid first-step 0.5 h infusion and slow second-step 3 h infusion); q8h, every 8 hours; q6h, every 6 hours; PTA, probability of target attainment; CFR, cumulative fraction of response. Bold values means PTA > 90% when MIC of AZA ≥ 16 mg/L.

## Discussion

Avibactam is currently marketed in combination with ceftazidime and has demonstrated high rates of activity against serine carbapenemases, but are ineffective against MBL (Vasoo et al., 2015). In contrast to CZA, AZA restores the activity of aztreonam against MBL-producing CRE via inhibition of coexpressed serine carbapenemases (Crandon and Nicolau, 2013; Cornely et al., 2020). Therefore, CZA represented remarkable advance and AZA represented the novel last defense in the treatments of infections caused by BSIs-CRKP. In this investigation, we assessed the *in vitro* activity and optimized treatments of CZA and AZA against 318 BSIs-CRKP isolates in China. The results showed ST11 KPC-producing BSIs-CRKP remained the major type. The susceptibility and CFR of AZA was higher than CZA against BSIs-CRKP. In addition, TSIT of CZA and AZA could improve the clinical effect against BSIs-CRKP.

Previous studies demonstrated CZA had higher rates of clinical success and survival than other regimens (Shields et al., 2017; van Duin et al., 2018). However, development of resistance has been reported in patients that received CZA monotherapy (Venditti et al., 2019; Ackley et al., 2020). In addition, the resistance rate to CZA was increasing. Recent data showed the resistance rate of CRKP to CZA reached 16.7–21%, consistent with our study (Wang et al., 2020). Therefore, optimal management of CZA against BSIs-CRKP, especially for KPC-producing isolates, has important clinical significance to treatment. As we know, CZA is usually recommended to administer as TIT of 2 g q8h. The MCS results revealed the CFR for CZA of TSIT was higher than TIT and PIT. In addition, the CFR of CZA 2 g 0.5 h, 3 g q8h 3 h infusion and CZA 3 g 0.5 h, 2 g q8h 3 h infusion reached ≥89%. So far, only one case reported a dose of CZA 2 g 2 h infusion q8h was appropriate for critically ill patient with *Pseudomonas aeruginosa* pneumonia and undergoing continuous venovenous hemodiafiltration (Soukup et al., 2019). Recently, a retrospective cohort study demonstrated TSIT (loading dose of 2.5 g 2 h infusion, then initial dosage adjustment depending on renal function every 12 h) of CZA achieved high clinical and microbiological cure rates against multidrug-resistant *P. aeruginosa* and *K. pneumoniae* infections (Goncette et al., 2021). However, there were only 10 patients in this study. Hence, based on our data and previous studies, further larger studies are warranted to confirm the clinical use of TSIT for CZA.

AZA is expected to have more utility in geographical regions where CRKP predominate, especially for MBL-producing isolates (Chew et al., 2018). Similar to previous surveillance data, our results showed higher susceptibility of AZA against BSIs-CRKP than CZA (Sader et al., 2021). This is largely due to aztreonam relatively stable against hydrolysis by MBL, making AZA as a unique potential treatment option for MBL- or serine carbapenemases-producing BSIs-CRKP (Crandon and Nicolau, 2013). Several studies supported the clinical development of AZA against complicated infections caused by CRE (Crandon and Nicolau, 2013; Biagi et al., 2019; Cornely et al., 2020). Phase 2a study of AZA confirmed TSIT (loading dose 0.5 g 0.5 h, 1.5 g q6h 3 h infusion) was appropriate for Phase 3 clinical trial (Cornely et al., 2020). Similarly, all simulated TSIT of AZA in our study achieved >96% of CFR. Therefore, these results from supported the clinical development of TSIT for AZA.

The method of antibiotics administration is of greater importance for treatment of bacteria with elevated MIC. In general, time-dependent antibiotics could attain a high peak concentration during TIT, while TIT also leads to precipitous drops due to short half-life (Tamma et al., 2011). Several real-world evidences have revealed that the PIT of β-lactams over TIT in terms of achieving more consistent serum concentration and aggressive %fT > MIC (Hanes et al., 2000; Jaruratanasirikul et al., 2005; Tamma et al., 2011). However, meta-analysis results found PIT of β-lactams were not associated with clinical advantage compared to TIT (Tamma et al., 2011; Shiu et al., 2013). The similar phenomenon occurred in MCS of CZA and AZA to evaluate CFR. Decrease in peak concentration and delay in time of peak concentration may be underlying causes of reduce of initial bactericidal effect during PIT. For solving these problems, TSIT has been adopted due to sufficient %fT > MIC and peak concentration, and short time of peak concentration (Eguchi et al., 2010). The CFR of CZA and AZA using TSIT was higher than TIT and PIT in our study as well. In addition, previous studies have demonstrated TSIP of carbapenems has been proven to have better initial bactericidal effects than PIT (Eguchi et al., 2010; Huang et al., 2020). Therefore, it is expected that the therapeutic effect of CZA and AZA against BSIs-CRKP will be further enhanced by TSIT in comparison with TIT or PIT.

To our knowledge, our study is the first to optimize the treatments of CZA and AZA against BSIs-CRKP in China. However, there are several limitations in our study as well. First, the included hospitals surveyed in each geographic region were limited. Only one hospital participated in NE, NW, and SW, respectively. Second, most BSIs-CRKP isolates carried *bla*_KPC_ gene. Third, the *in vitro* activity and simulation could not evaluate the host immune response. Further randomized clinical trials are urgently needed to evaluate the efficacy and safety of CZA and AZA.

## Conclusion

Clonal expansion of ST11 remained responsible for the dissemination of KPC-producing BSIs-CRKP. AZA exhibited highly potent *in vitro* activity against BSIs-CRKP. In addition, TSIT of CZA and AZA provided higher CFR than TIT or PIT. Therefore, a switch to TSIT may improve clinical outcomes in patients caused by BSIs-CRKP.

## Data Availability

The original contributions presented in the study are included in the article/Supplementary Material, further inquiries can be directed to the corresponding authors.
