# Supplementary figures and images for: Antibacterial Activity and Optimal Treatment of Ceftazidime-Avibactam and Aztreonam-Avibactam Against Bloodstream Infections Caused by Carbapenem-Resistant Klebsiella pneumoniae
Source: Front Pharmacol. 2021 Dec 14;12:771910. doi: 10.3389/fphar.2021.771910 (PMC8712734; doi:10.3389/fphar.2021.771910)

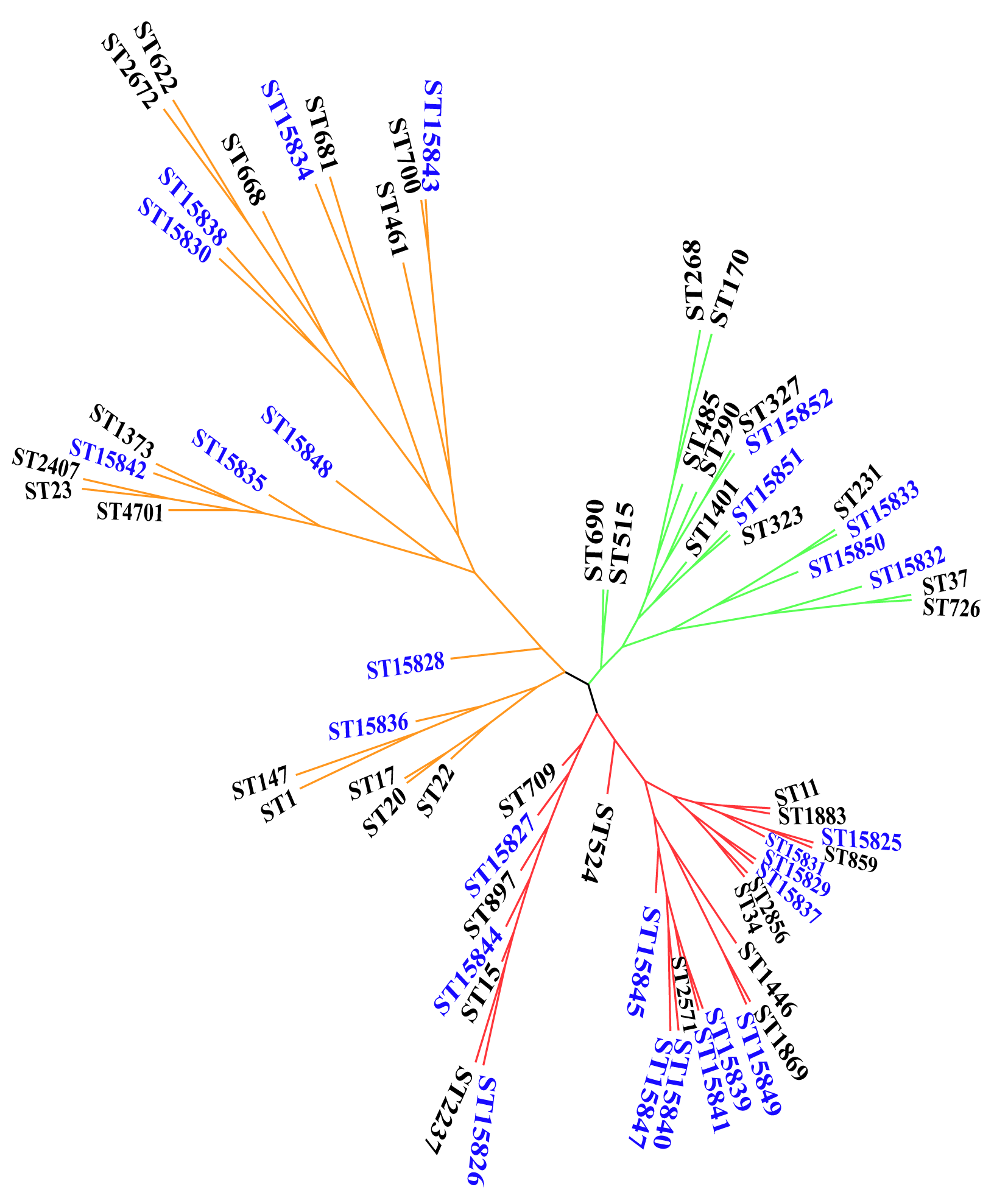

Supplement: Supplementary file 1 [file Image2.TIF]

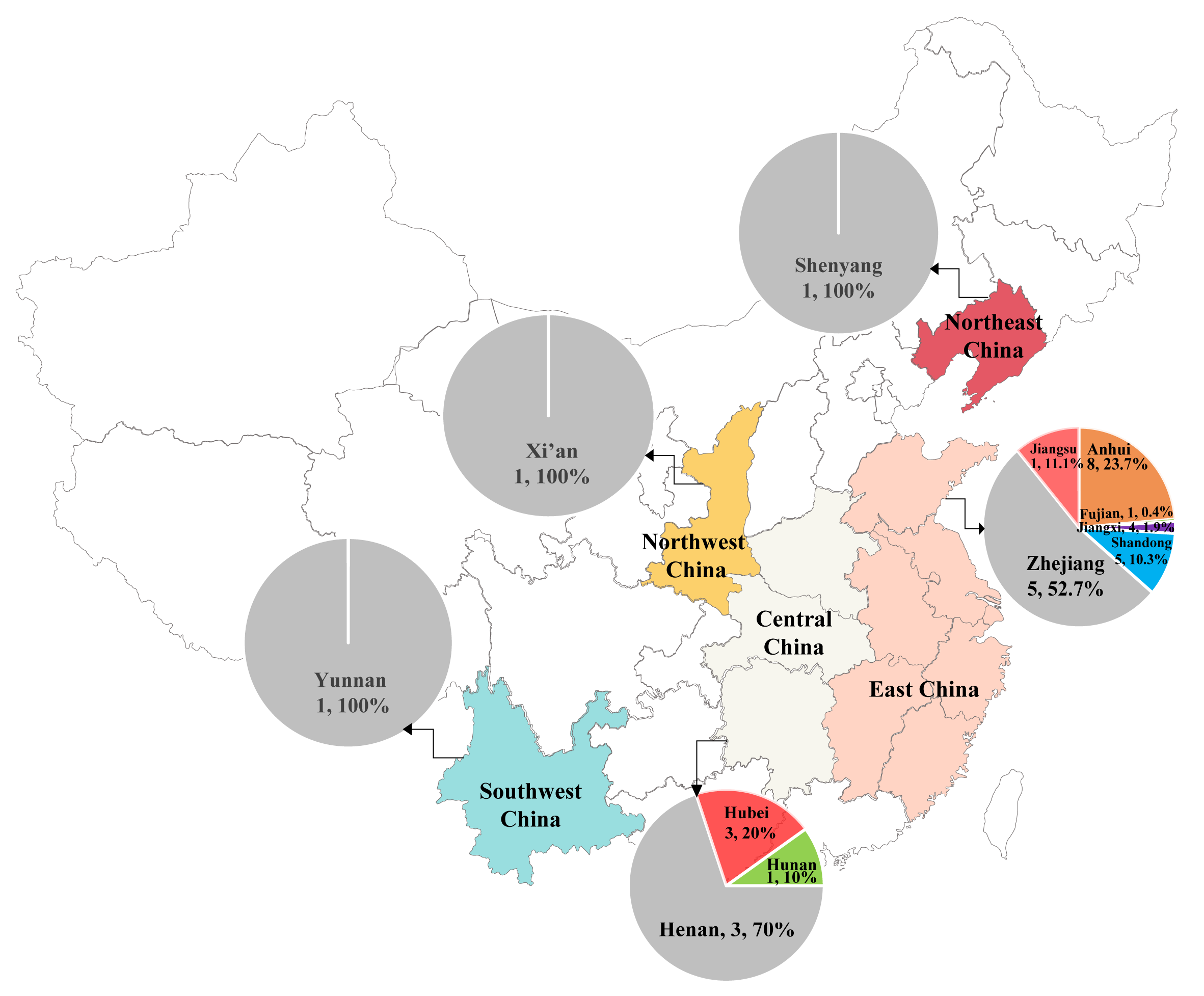

Supplement: Supplementary file 2 [file Image1.TIF]
