# Supplementary material for: Antibacterial Activity and Optimal Treatment of Ceftazidime-Avibactam and Aztreonam-Avibactam Against Bloodstream Infections Caused by Carbapenem-Resistant Klebsiella pneumoniae
Source: Front Pharmacol. 2021 Dec 14;12:771910. doi: 10.3389/fphar.2021.771910 (PMC8712734; doi:10.3389/fphar.2021.771910)
Supplement: Supplementary file 3 [file DataSheet1.docx]

**Supplementary Table 1.** Primers used in detecting MLST

| **Name of primers** | **Sequence (5’-3’)** | | | **Length (bp)** |
| --- | --- | --- | --- | --- |
| rpoB-F | GGCGAAATGGCWGAGAACCA | | | 501 |
| rpoB-R | GAGTCTTCGAAGTTGTAACC | | |  |
| gapA-F | TGAAATATGACTCCACTCACGG | | | 450 |
| gapA-R | CTTCAGAAGCGGCTTTGATGGCTT | | |  |
| mdh-F | CCCAACTCGCTTCAGGTTCAG | | | 477 |
| mdh-R | CCGTTTTTCCCCAGCAGCAG | | |  |
| pgi-F | GAGAAAAACCTGCCTGTACTGCTGGC | | | 432 |
| pgi-R | CGCGCCACGCTTTATAGCGGTTAAT | | |  |
| phoE-F | ACCTACCGCAACACCGACTTCTTCGG | | | 420 |
| phoE-R | | TGATCAGAACTGGTAGGTGAT |  | |
| infB-F | | CTCGCTGCTGGACTATATTCG | 318 | |
| infB-R | | CGCTTTCAGCTCAAGAACTTC |  | |
| ton-F | | CTTTATACCTCGGTACATCAGGTT | 414 | |
| ton-R | | ATTCGCCGGCTGRGCRGAGAG |  | |

**Supplementary Table 2.** Equations for %fT> MIC

| **Infusion**  **methods** | **Equations** |
| --- | --- |
| **TIT** | $\%fT>MIC=Ln(\frac{Dose*fu}{V_{\text{d}}*MIC})*\frac{V_{d}}{CL}*\frac{100}{DI}$ |
| **PIT** | $fT>MIC=[T_{INF}-[Ln[\frac{R_{0}/CL}{R_{0}/C\text{L}-MIC}]*\frac{V_{d}}{CL}]]+[Ln[\frac{R_{0}/CL-R_{0}/CL*e^{(-CL/VdT_{INF})}}{MIC}]*\frac{V_{d}}{CL}]$ $\%fT>MIC=fT>MIC*100/DI$   |
| **TSIT** | (a) $fT>MIC=T_{1}+(Ln\frac{R_{01}(1-\exp(-CL*T_{1}/V_{d}))-R_{02}}{CL*MIC-R_{02}})*\frac{V_{d}}{CL}-(Ln\frac{R_{01}}{R_{01}-CL*MIC})*\frac{V_{d}}{CL}$ |
|  | (b) $fT>MIC=T_{1}+T_{2}+(Ln\frac{R_{02}+(R_{01}-R_{02})\exp(-CL*T_{2}/V)-R_{01}\exp(-CL*(T_{1}+T_{2})/V_{d}}{CL*MIC})*\frac{V_{d}}{CL}-(Ln\frac{R_{01}}{R_{01}-CL*MIC})*\frac{V_{d}}{CL}$ $\%fT>MIC=fT>MIC*100/DI$   |

TIT, traditional 0.5h infusion therapy; PIT, prolonged 3h infusion therapy; TSIT, two-step infusion therapy (rapid first-step 0.5h infusion and slow second-step 3h infusion); MIC, minimum inhibitory concentration (mg/L); Ln, natural logarithm; Dose, intermittent dose (mg); fu, free drug fraction; V_d_, volume of distribution (L); CL, clearance rate (L/h); DI, dosing interval (h); T_INF_, prolonged infusion time (h); R_0_, prolonged infusion rate (mg/h); e, natural constant; T_1_, infusion time of the first step (h); T_2_, infusion time of the second step (h); R_01_, first-step infusion rate (mg/h); R_02_, second-step infusion rate (mg/h); exp, exponent; (a) If the concentration of CZA or AZA falls to the MIC level during the second step of infusion; (b) If the concentration of CZA or AZA falls to the MIC level after the second step of infusion is terminated.
